# Epigenetic Control of Alveolar Macrophages: Impact on Lung Health and Disease

**DOI:** 10.3390/cells14090640

**Published:** 2025-04-25

**Authors:** Nirmal Parajuli, Kalpana Subedi, Xzaviar Kaymar Solone, Aimin Jiang, Li Zhou, Qing-Sheng Mi

**Affiliations:** 1 Center for Cutaneous Biology and Immunology Research, Department of Dermatology, Henry Ford Health, Detroit, MI 48202, USA; nparaju1@hfhs.org (N.P.); ksubedi1@hfhs.org (K.S.); xsolone1@hfhs.org (X.K.S.); ajiang3@hfhs.org (A.J.); 2 Immunology Research Program, Henry Ford Cancer Institute, Henry Ford Health, Detroit, MI 48202, USA; 3 Department of Medicine, College of Human Medicine, Michigan State University, East Lansing, MI 48824, USA; 4 Department of Medicine, Henry Ford Health, Detroit, MI 48202, USA

**Keywords:** alveolar macrophage, epigenetics, histone modification, DNA methylation

## Abstract

Alveolar macrophages (AMs) are immune cells located in the alveoli—the tiny air sacs in the lungs where gas exchange occurs. Their functions are regulated by various epigenetic mechanisms, which are essential for both healthy lung function and disease development. In the lung’s microenvironment, AMs play critical roles in immune surveillance, pathogen clearance, and tissue repair. This review examines how epigenetic regulation influences AM functions and their involvement in lung diseases. Key mechanisms, such as DNA methylation, histone modifications, and non-coding RNAs, regulate gene expression in response to environmental signals. In healthy lungs, these modifications enable AMs to quickly respond to inhaled threats. However, when these processes malfunction, they could contribute to diseases such as pulmonary fibrosis, COPD, and pulmonary hypertension. By exploring how epigenetic changes affect AM polarization, plasticity, and immune responses, we can gain deeper insights into their role in lung diseases and open new avenues for treating and preventing respiratory conditions. Ultimately, understanding the epigenetic mechanisms within AMs enhances our knowledge of lung immunology and offers potential for innovative interventions to restore lung health and prevent respiratory diseases.

## 1. Introduction

**Figure 1:**
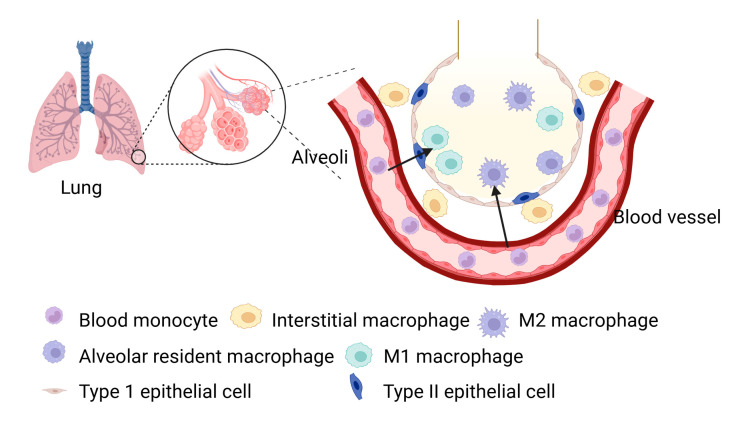
Anatomic localization of various macrophages within the alveoli of normal lung.

Macrophages are essential components of the innate immune system, functioning as phagocytes that utilize pattern recognition receptors to detect and eliminate pathogens and debris [1,2]. In the lungs, two primary populations of macrophages exist: alveolar resident macrophages (AMs) and interstitial macrophages (IMs) [3]. AMs are mainly located in the alveoli, where they serve as the first line of defense, protecting the respiratory system from inhaled pathogens and particles to maintain lung homeostasis [4,5]. In contrast, IMs reside in the tissue surrounding the alveoli, playing a critical role in tissue repair during injury, infection, or inflammation [6,7] (Figure 1). These macrophages can adopt either pro-inflammatory or anti-inflammatory phenotypes depending on the signals they receive, which is reflected in the expression of markers such as CD11c, CD11b, and various colony-stimulating factor receptor subtypes [8].

## 2. Alveolar Macrophages in Mice

**Figure 2:**
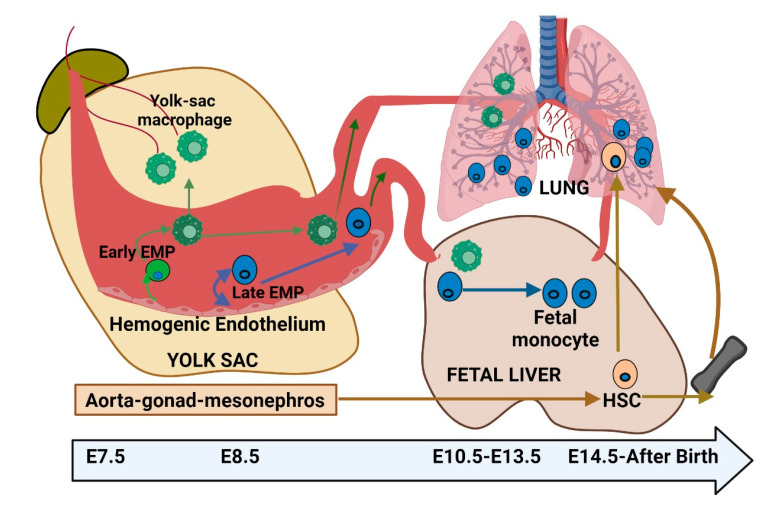
Embryonic development of lung alveolar macrophages—insights into origin and differentiation.

During embryonic development, resident AMs originate from the yolk sac (YS) and fetal liver (FL), establishing a population of self-renewing cells [9,10]. In contrast, IMs have a more complex origin, arising from both YS-derived macrophages and bone marrow (BM)-derived monocytes. The development of resident AMs in mice is a fascinating process that occurs in multiple stages, spanning both embryonic and postnatal phases. During early embryogenesis, around embryonic day 9.5, YS-derived macrophages are among the first immune cells to colonize the developing lungs [11,12]. These macrophages play a critical role in the early establishment of the immune system within the lung tissue. As development progresses, around embryonic day 12.5, fetal liver-derived monocytes contribute to the growing AM population [13] (Figure 2). These monocytes migrate to the lungs, where they differentiate into mature AMs. The coordination of various signaling pathways and transcription factors, such as *PU.1*, is crucial in guiding these developmental processes [8,14]. After birth, the lungs continue to undergo significant changes, and the AM population becomes more firmly established. By approximately postnatal day 10, a well-defined and functional AM population emerges from both YS and FL precursors. The migration, seeding, and differentiation of these macrophages are primarily regulated by the colony-stimulating factor 1 receptor (CSF-1R) and sustained through the recruitment of circulating monocytes via the CCL2/CCR2 axis [15]. These macrophages exhibit a remarkable capacity for replenishment by circulating monocytes and are categorized into three distinct subpopulations: IM1, IM2, and IM3, based on specific surface markers [10,16,17]. Once established, AMs play an essential role in immune surveillance within the lung tissue [12].

## 3. Alveolar Macrophages in Humans

Unlike in mice, understanding the origin and development of human lung AMs, particularly in the context of lung diseases, is limited due to the impracticality of invasive in vivo experiments. However, valuable insights have emerged from studies involving BM and lung transplantations. Allogeneic BM transplantation depletes host AMs and results in rapid turnover and mixed host–donor chimerism, highlighting the role of donor hematopoiesis in replenishing AMs. In contrast, lung transplantation preserves lung macrophages, allowing donor-derived AMs in persistence [12,18]. The varying extent of donor AM persistence suggests that human AMs are primarily maintained through local self-renewal in the steady-state lung. During lung injury with macrophage depletion, monocytes replace resident AMs. The presence of AMs in certain leukemias, despite depleted blood monocytes, suggests that AMs may be independent of circulating monocytes under steady-state conditions [12]. However, the embryonic origin of human AMs in the developing lung remains uncertain, and the specific progenitors responsible for their development, whether they are embryonic or adult, are still elusive.

All lung macrophages, as essential components of the pulmonary immune system, undergo complex epigenetic changes that carefully regulate their functions in both health and disease [19,20]. Epigenetics plays a critical role in shaping the behavior of lung macrophages, which display remarkable plasticity, allowing them to adapt their responses to a variety of environmental cues and precisely adjust their epigenetic marks [20,21,22]. This review explores the complex role of epigenetics in regulating gene expression, plasticity, and adaptability in AMs, highlighting how mechanisms such as DNA methylation, histone modifications, non-coding RNAs, and epigenetic inheritance are crucial for modulating AM responses to environmental signals, both in normal lung homeostasis and disease states. By examining current research, it also aims to provide a comprehensive overview of how these mechanisms influence AM function and identify potential therapeutic strategies for manipulating AM activity in lung diseases.

## 4. Epigenetic Modification in Lung Macrophages

Epigenetics controls gene expression without altering the DNA sequence, influencing cell identity through chromatin structure and DNA accessibility [23,24,25]. While once considered stable, epigenetic markers are now known to change in response to development and environmental signals [26]. These changes involve mechanisms like DNA methylation, histone modifications, and non-coding RNAs, all of which regulate gene expression by affecting protein–DNA interactions and mRNA stability.

### 4.1. DNA Methylation

**Figure 3:**
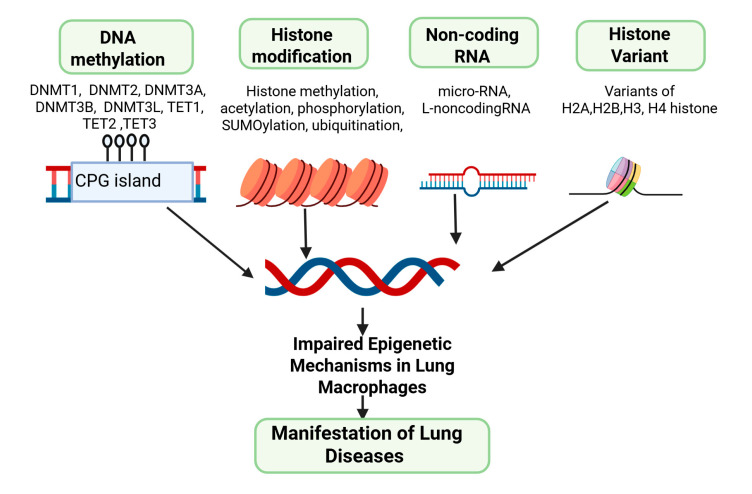
Illustrative role of epigenetic mechanisms within lung macrophages for manifesting lung disease.

DNA methylation is a crucial epigenetic modification that regulates gene expression in lung macrophages [27], influencing their immune roles [28,29]. This process involves adding methyl groups to specific cytosine residues in DNA, typically at CpG dinucleotides. DNA methylation is catalyzed by DNA methyltransferases (Dnmts), including *Dnmt1*, *Dnmt2*, *Dnmt3a*, *Dnmt3b*, and *Dnmt3l* [30,31]. Additionally, the methylated cytosine can be converted to 5-hydroxymethylcytosine by Tet methylcytosine dioxygenases (Tets), such as *Tet1*, *Tet2*, and *Tet3* (Figure 3). The primary function of DNA methylation in lung macrophages is to fine-tune their response to various stimuli, crucially regulating immune functions [32]. Typically, DNA methylation leads to gene repression by blocking transcription factor binding and RNA polymerase activity when a methyl group is added to a CpG site in the gene’s promoter region [33]. This inhibition is essential for silencing genes that are not needed under certain conditions [34]. During infection, immune genes may undergo demethylation, enabling their rapid expression and enhancing the immune response [29]. Conversely, during immune quiescence, these genes may be remethylated to maintain their silenced state [35].

Furthermore, DNA methylation provides lung macrophages with a form of epigenetic memory, marking previous exposures to pathogens or environmental factors [30,36]. This memory shapes their response when encountering the same stimulus again, allowing for a more effective reaction to recurring infections [30]. External factors such as diet, pollutants, and oxidative stress can alter DNA methylation patterns in lung macrophages [37]. As macrophages age, their DNA methylation patterns shift, impacting their functions and response to infections and diseases [38]. These age-related changes are particularly significant in conditions like idiopathic pulmonary fibrosis (IPF), where aging is a known risk factor [38]. Alterations in DNA methylation at cytosine–guanine dinucleotides affect chromatin accessibility, transcription factor binding, and gene expression in AMs. As one of the most stable epigenetic markers, DNA methylation serves as a measurable indicator of how development and disease influence the epigenome.

In lung macrophages, DNA methylation is modified in chronic airway diseases, driven by both genetic and environmental factors [39]. An extensive epigenome-wide analysis has identified 95 differentially methylated CpGs in lung macrophages across different lung regions, from the upper to lower lobes [28]. This finding suggests that regional variations in macrophage metabolism may contribute to distinct immune responses in different parts of the lung [28,34]. The anatomical differences in the lung influencing DNA methylation patterns in AMs highlights the role of the lung microenvironment in shaping AM development [29]. Understanding these epigenetic processes is crucial for advancing lung immunology and developing targeted treatments for lung diseases. However, despite recent progress in understanding the ontogeny of lung macrophages, our knowledge of the epigenetic mechanisms driving the transition from monocytes to macrophages in the lung and how diseases impact these processes remains limited.

### 4.2. Histone Modification

Histone modifications involve a class of proteins that play a fundamental role in the packaging of DNA into chromatin and the regulation of gene expression [40]. These processes are crucial epigenetic mechanisms that influence the function and responses of lung macrophages [41]. Histones play a central role in packaging DNA into chromatin and regulating gene expression [40,42] (Figure 3). Modifications of histones including methylation, acetylation, phosphorylation, ubiquitylation, and sumoylation involve covalent changes to histone proteins, significantly affecting gene expression. By altering the chromatin structure and interacting with histone modifiers, these modifications affect various biological processes.

Histone acetylation and deacetylation are vital processes in gene regulation, typically catalyzed by enzymes with histone acetyltransferase (HAT) or histone deacetylase (HDAC) activity. Acetylation involves the transfer of an acetyl group from acetyl coenzyme A to a target molecule, while deacetylation is the reverse process, removing the acetyl group [43]. These modifications are particularly important in macrophages, where specific histone methylation patterns help prevent excessive inflammation by restricting access to pro-inflammatory genes. In general, histone H3 can be acetylated at lys 9, 14, 18, 23, and 56, methylated at arginine 2 and lys 4, 9, 27, 36, and 79, and phosphorylated at ser 10, 28, and Thr 3, 11. Similarly, histone H4 undergoes acetylation at lys 5, 8, 12, and 16, methylation at arginine 3 and lys20, and phosphorylation at Ser 141 and 142. These modifications are essential for regulating transcription, chromatin packaging, and DNA repair. In lung macrophages, these modifications play a pivotal role in activating immune response genes when encountering pathogens [44]. For instance, histone acetylation is associated with enhanced gene expression [45]. Conversely, histone methylation can lead to chromatin condensation, suppressing gene expression [44].

Among the various HDAC enzymes, HDAC3 deficiency in AMs leads to severe mitochondrial oxidative dysfunction and cell death by impairing *Ppar*γ expression, including its downstream signaling pathways [46]. Interestingly, HDAC3-deficient macrophages alleviate LPS-induced acute lung injury [46] and reduce pyroptosis in murine lung tissues by decreasing histone acetylation. These findings suggest that histone modifications play a significant role in lung injury [42,46,47,48,49]. In summary, histone modifications play a pivotal role in governing the function and responses of AMs, with far-reaching implications for lung health and disease susceptibility. Understanding these mechanisms is essential for not only advancing our knowledge of lung immunology but also developing targeted interventions for respiratory conditions.

### 4.3. Non-Coding RNAs

Non-coding RNAs (ncRNAs) are microRNAs and long non-coding RNAs (lncRNAs) that modulate gene expression by interacting with the epigenetic machinery [50,51]. NcRNAs are involved in regulating gene expression and chromatin structure, thereby impacting the functions and responses of these immune cells [50,52]. Additionally, emerging evidence suggests that lncRNAs play a role in regulating macrophage polarization, exerting consequential effects on the manifestation of respiratory diseases [53,54]. MicroRNAs are short RNA molecules that bind to mRNAs, leading to mRNA degradation or translational repression [55]. In AMs, specific microRNAs including *miRNA-124*, *miRNA-155*, and *miRNA-223* can target genes involved in immune responses and inflammation, effectively shaping the immune function of these cells [56]. Moreover, these microRNAs play a crucial role in regulating lung inflammation and directing macrophage polarization [57,58]. Elevated expression of *miRNA-124* suppresses M1 macrophage activity, whereas *miRNA-155* promotes M1 polarization. Conversely, depletion of *miRNA-223* leads to M1 polarization [59]. Certainly, the nuanced regulatory role of microRNAs is evident in both lung inflammation and macrophage functions.

Likewise, lncRNAs are longer RNA molecules that interact with chromatin and epigenetic modifiers, affecting chromatin structure and gene accessibility [60]. They either enhance or repress gene expression, depending on the context. In AMs, lncRNAs may be involved in regulating immune genes and determining the macrophage’s regulatory response to various stimuli. These non-coding RNAs also contribute to epigenetic memory in AMs [53] and exert a lasting impact on the cell’s ability to respond to recurring challenges by influencing the expression of genes involved in immune responses [54] (Figure 3). Understanding the role of non-coding RNAs in AMs, epigenetic regulation is essential for unraveling the intricacies of lung immunology and developing targeted interventions for respiratory conditions.

### 4.4. Histone Variants

Histone variants are specialized variants of histone proteins with unique amino acid sequences, playing integral roles in orchestrating chromatin structure and gene expression. Unlike the canonical histones, these specialized histone proteins are incorporated into nucleosomes by replacing canonical histones and contribute to the structural and functional diversity of chromatin.

Histones are categorized into two primary groups: canonical histones and histone variants [61]. Canonical histones are the core histones that form the structural backbone of the nucleosome, which is the basic repeating unit of chromatin. There are five major types of core canonical histones notably known for their individual function, including histone 2A (H2A) for stabilizing the nucleosome structure, histone 2B (H2B) for nucleosome stability, centrally located histone 3 (H3) for regulating gene expression through post-translation modification, and histone 4 (H4) for interacting with H3-mediated stabilization in the nucleosome structure, including its role in various post-translational modifications [61,62]. H2A, H2B, H3, and H4 form the nucleosome octamer while canonical linker histone 1 (H1) binds with the DNA between nucleosomes, promoting higher-order chromatin structure.

Among these variants, several have distinct isoforms, each with specific functions. In the H2A family, H2A.Z encompasses H2A.Z.1 and H2A.Z.2, both associated with gene activation by promoting an open chromatin structure. Meanwhile, H2A.X includes H2A.X.1 and H2A.X.2, critical for DNA repair by becoming phosphorylated at DNA double-strand breaks [62,63]. In the H2B category, H2B.Z.1 and H2B.Z.2 are associated with active gene transcription and nucleosome stability. Among the H3 variants, H3.3 comprises H3.3A and H3.3B, linked to gene regulation and epigenetic memory [61,62,63]. CENP-A, another H3 variant, has multiple isoforms crucial for centromere formation [64]. In the H4 family, various isoforms of H4K20me1 are involved in gene silencing and heterochromatin formation. Each of these histone variants and their isoforms adds depth to the regulation of chromatin structure and gene expression, playing vital roles in various biological processes and genomic stability [63] (Figure 3). Nevertheless, a defined role for any histone variant specifically designed to comprehend its impact on AMs has not been established. Our current research is directed towards uncovering this aspect. Hence, understanding the complexities of these histone variants and their isoforms is essential for unraveling the intricacies of epigenetic regulation in AMs.

Overall, the interconnectedness of epigenetic mechanisms in AMs reveals a sophisticated regulatory network that enables these immune cells to finely tune their responses to the constantly changing lung environment. A foundational aspect of this integration lies in the interplay between DNA methylation and histone modifications [26,65,66]. DNA methylation, often associated with stable gene silencing, can act for specific protein complexes; for instance, MBD proteins not only recognize methylated DNA but also recruit histone-modifying enzymes such as HDACs, thereby linking DNA methylation to chromatin compaction and transcriptional repression [67,68]. Conversely, certain histone modifications can influence DNA methylation. For example, H3K4me3, commonly found at actively transcribed gene promoters, can inhibit the binding and activity of DNMTs, maintaining these regions in a transcriptionally permissive state [69]. Likewise, the co-occurrence of histone acetylation, typically linked to gene activation, with certain histone methylation marks can establish a unique chromatin environment that either enhances or represses gene expression, depending on the precise modifications and their genomic context [70]. Adding further nuance is the emerging field of metabolic epigenetics, which highlights how fluctuations in cellular metabolism directly impact epigenetic enzyme activity. Metabolites such as S-adenosylmethionine (SAM), derived from methionine metabolism, serve as essential methyl donors for DNMTs and some HMTs, meaning changes in methionine availability can significantly alter methylation landscapes [71]. Similarly, acetyl-CoA, a central metabolite in energy production, provides the acetyl group for HATs, so shifts in glucose and fatty acid metabolism can dynamically influence histone acetylation and gene expression [72].

These interconnected mechanisms become particularly relevant in various disease states [73], including pulmonary fibrosis [65]. In AMs, when transitioning toward a profibrotic (M2-like) phenotype, aberrant DNA methylation patterns may silence genes involved in inflammation resolution or extracellular matrix (ECM) degradation while simultaneously fostering a chromatin landscape that is receptive to activating epigenetic marks [74]. Moreover, the heightened glycolytic metabolism characteristic of pro-inflammatory and profibrotic macrophages leads to increased production of lactate, which serves as the substrate for histone lactylation [75,76], a recently discovered modification that promotes the expression of genes implicated in ECM production and inflammatory signaling. It is plausible that specific DNA methylation patterns may prime certain genomic regions for activation by lactylation, or that lactylation itself stabilizes transcriptional programs initiated by DNA methylation changes. Understanding these synergistic interactions, where metabolic reprogramming fuels epigenetic modifications that reinforce pathological phenotypes, offers a promising avenue for therapeutic intervention. By targeting and disrupting these interconnected pathways, it may be possible to reprogram AMs toward a more inflammation-resolving, fibrosis-limiting phenotype, offering potential treatments for pulmonary fibrosis and other lung diseases.

## 5. Epigenetic Regulation of Macrophages in Lung Diseases

Macrophage expansion and repopulation in response to injury, infection, or inflammation are tightly regulated and depend on the nature of the stimulus [3]. During acute inflammation or severe infection, AMs are temporarily lost [77]. For example, in mice, sterile inflammation induces the transient loss of AMs, followed by the expansion of IMs within lung tissue [78]. This IM expansion is blocked in the absence of CCR2, emphasizing the importance of monocyte recruitment in this process. In contrast, during the resolution of inflammation, the repopulation of AMs primarily depends on local proliferation [3]. However, in more severe inflammatory conditions, such as lung fibrosis, resident AMs are often replaced by monocyte-derived macrophages [6]. It remains unclear whether this replacement is due to severe inflammation affecting the self-renewal of AMs or is driven by structural changes. Furthermore, classical monocytes can migrate into the airways in response to injury or infection [3,21]. There is also evidence suggesting that monocyte-derived macrophages in the interstitial space can migrate to the airways. However, the extent to which these alternative differentiation pathways influence the fate and function of macrophages remains uncertain, highlighting the need for further research into the complex dynamics of macrophage responses in different inflammatory contexts.

Resident AMs showed remarkable plasticity, shifting from pro-inflammatory (M1) to anti-inflammatory/tissue-repair (M2) phenotypes, and they are largely controlled by epigenetic mechanisms that alter gene expression without changing the DNA sequence [47,77,79,80]. These epigenetic changes in lung macrophages modulate inflammation, immune tolerance, tissue remodeling, and disease progression in various lung diseases [47,80]. Understanding this process sheds light on how the epigenetic regulation of AMs contributes to the pathogenesis of conditions such as pulmonary fibrosis, chronic obstructive pulmonary disease, lung cancer, and other respiratory disease conditions.

### 5.1. Lung Fibrosis

Idiopathic pulmonary fibrosis (IPF) is a progressive lung disease characterized by the scarring of lung tissue, which leads to impaired lung function [81]. Although the exact cause of IPF is unknown, factors such as genetics, environmental exposures, and abnormal immune responses are believed to contribute to its development [82,83]. Additionally, macrophages play a key role in IPF by becoming activated in response to lung injury. Once activated, they adopt profibrotic functions by secreting growth factors (such as TGF-β and PDGF), proteases, and chemokines that recruit fibroblasts and induce extracellular matrix deposition, contributing to lung scarring [84,85]. Their involvement in pulmonary fibrosis includes the depletion of resident AMs, increase in IMs, and initiation and sustainment of the inflammatory response within lung tissue [86,87]. Indeed, resident AMs participate in resolving inflammation and aiding tissue repair through the phagocytosis of cellular debris and apoptotic cells in response to lung injury [87,88]. Dysregulated macrophage activity, along with their interactions with fibroblasts, exacerbates chronic inflammation and excessive tissue repair, both of which drive disease progression.

#### 5.1.1. DNA Methylation

Recent high-resolution studies have mapped the DNA methylome of AMs in IPF patients [29]. McErlean et al. demonstrated that the AMs of IPF patients exhibit distinct methylation signatures compared with healthy controls, suggesting widespread epigenetic reprogramming during fibrosis [29,32]. Many of the methylated altered CpG sites in IPF AMs are in intronic or intergenic regions associated with open chromatin. This suggests a functional link between methylation changes and gene regulation [26]. Interestingly, while IPF is an age-related disease and global DNA methylation generally correlates with age [89,90], IPF AMs did not show a methylation pattern like accelerated aging [29]. Instead, specific pathways were affected, with IPF AMs including differential methylation in genes related to lipid and glucose metabolism [29,32]. This aligns with the concept of macrophage immunometabolism in fibrosis, where changes in the methylation of key metabolic regulators can push AMs toward a profibrotic metabolic state, such as Warburg-like glycolysis, which supports fibrotic activation [29]. Overall, DNA methylation profiles contribute to the altered phenotype of AMs in fibrosis.

Macrophages display diverse phenotypes in pulmonary fibrosis, with M1 macrophages being linked to inflammation and fibrosis [86] and M2 macrophages contributing to tissue repair and anti-inflammatory functions [91]. The balance between M1 and M2 macrophages plays a key role in fibrosis progression [91]. Research has highlighted the role of monocyte-derived M2 macrophages in bleomycin-induced fibrosis, where they replace resident AMs, provoke inflammatory responses, and contribute to lung fibrosis by releasing profibrotic factors [87]. The role of IMs in lung fibrosis is gradually becoming clearer, though their precise function remains unclear. Both IMs and resident AMs are present in clinical and preclinical models of radiation-induced lung fibrosis [3,10]. Interestingly, depleting IMs using a colony-stimulating factor receptor-1 neutralizing antibody effectively reduces fibrosis in vivo, while depletion of tissue-resident AMs does not impact the fibrosis score [87]. AMs during fibrosis often exhibit an M2-like activation state which, as mentioned, is influenced by both DNA methylation and specific histone modifications [92]. While it is well established that DNA methylation contributes to the pathogenesis of IPF, how the expression and activity of DNA methyltransferase family members influences IPF is not entirely understood. In one study, higher DNMT3a and DNMT3b expression were found in patients with IPF relative to levels in normal control subjects [93], but no significant difference was observed in DNMT1 expression, suggesting that DNMTs may be upregulated in IPF [32,90,92,93].

In fibrotic animal models, the number of M2 macrophages, including the expression of profibrotic markers, were increased in myeloid lineage-specific DNMT3B-deficient mice challenged with bleomycin compared with controls [94]. This increased M2 polarization in myeloid DNMT3B-deficient mice was accompanied by increased pulmonary fibrosis, which suggests that macrophage DNMT3B inhibits the development of pulmonary fibrosis by restricting alternative macrophage polarization [92]. Given the importance of macrophages in the development of pulmonary fibrosis and the role of DNMT3B in macrophage polarization, myeloid DNMT3B limits the development of pulmonary fibrosis by limiting M2 macrophage polarization potentially by methylation of the promoter of the gene encoding Arg1 [29,87,93]. Profiling these DNA methylation from healthy subjects and IPF patients showed that aberrant macrophage polarization played a crucial role in developing IPF [29,30,93]. During the development of IPF, DNMTs are associated with inappropriate gene expression activation, resulting in the opposite roles of M1/M2 polarization [95]. These data suggest that DNA methylation mediated by different DNMTs may contribute to IPF pathogenesis [96]. The study showed that DNMT3B is crucial for chromosomal stability through DNA methylation and influences macrophage polarization, impacting the progression of IPF [97].

In the epigenetics domain, methyl-CpG-binding domain (MBD) proteins, particularly MBD2, regulate gene expression by binding to methylated DNA [96,98]. Increased MBD2 expression in IPF macrophages contributes to fibrosis, while its deficiency reduces collagen deposition and improves the fibrotic response through PI3K/Akt inhibition [99]. Similarly, the methyl-CpG-binding protein 2 (MECP2) influences profibrotic macrophage polarization, and targeting it with siRNA liposomes reduces fibrosis in mice [86]. Though DNA methylation changes are seen in IPF lungs, their exact role in macrophages remains unclear, with DNA methyltransferases playing a complex role in regulating M2 macrophage polarization in fibrosis [29].

#### 5.1.2. Histone Modification

In IPF, certain histone deacetylases are upregulated in the lung, potentially contributing to the disease. Notably, HDAC6 expression is increased in IPF lung tissues, and inhibiting HDAC6 has been shown to attenuate fibrosis [100,101]. HDAC6 deacetylates non-histone proteins like α-tubulin, and inhibiting it with compounds like trichostatin or tobramycin reduces TGF-β signaling and improves bleomycin-induced fibrosis in mice, suggesting that aberrant deacetylation in IPF AMs promotes fibrogenesis by enabling persistent activation of TGF-β target genes or by suppressing antifibrotic genes [100,102]. Conversely, histone acetylation marks, such as H3K4me3 and H3K9ac, are enriched in fibrotic macrophages [103]. A murine study showed that treating fibrotic lungs with the HDAC inhibitor valproic acid increased these marks at the IL-6 promoter, boosting an IL-6 response that paradoxically attenuated fibrosis by shifting macrophages away from a profibrotic M2 state [104,105,106]. Changes in histone modifications, particularly a reduction in acetylation, play a significant role in the development of fibrosis in IPF [107]. Various studies have indicated a deficiency in histone H3 and H4 acetylation at the promoter regions of genes associated with antifibrotic effects or apoptosis, such as cyclooxygenase 2, CXC chemokine gamma, IFN-y-inducible protein, and Fas, in fibroblasts from IPF patients [108,109]. These findings underscore the involvement of histone modification changes within macrophages in the pathogenesis of IPF [109]. Despite the success of histone deacetylase inhibitors in addressing fibrotic responses [110], the specific role of macrophage-specific histone modifications in lung fibrosis remains unclear. Additionally, profibrotic macrophages are linked to histone lactylation, driven by elevated lactate levels in fibrotic tissue [111]. Lactate produced by glycolytic macrophages and damaged cells can lactylate histone lysines and activate genes like *Arg1* and *VEGFA*, promoting tissue remodeling [112]. While lactylation was first noted in tumor macrophages [112,113], it likely occurs in IPF [114], with ongoing research exploring this novel epigenetic mark.

The epigenomic landscape of macrophages plays a key role in fibrosis [32]. Resident AMs and monocyte-derived AMs, which accumulate during injury, have distinct chromatin states, and both contribute to IPF [32]. In fibrosis, monocyte-derived AMs exhibit an epigenetic profile focused on tissue repair, with open chromatin at loci for *TGF-β*, *PDGF*, and scavenger receptors, and closed chromatin at pro-inflammatory cytokine loci [85,115]. This promotes fibroblast and epithelial interactions for matrix deposition instead of inflammation. Chromatin remodelers like *BRG1* of the *SWI/SNF* complex, which normally activate antifibrotic genes, are altered in fibrosis, leading to reduced *PPAR-γ* expression in AMs [116]. Without *PPAR-γ*, AMs release more TGF-β and fail to resolve fibrosis. These changes in chromatin configuration are central to macrophage-driven fibrosis [116].

#### 5.1.3. Non-Coding RNA

Next, non-coding RNA regulation of AMs has emerged as a key driver of pulmonary fibrosis. A notable example is *miR-33*, which is upregulated in AMs from IPF patients and regulates lipid metabolism [117]. Elevated *miR-33* suppresses genes involved in cholesterol efflux, fatty acid oxidation, and mitochondrial maintenance, promoting a dysfunctional, profibrotic macrophage phenotype. Deleting *miR-33* in mice protected against lung fibrosis, enhancing autophagy and mitochondrial health while reducing inflammatory cytokines. Inhibition of *miR-33* also reduced fibrosis in vivo [117]. These findings highlight miR-33 as an important regulator of macrophage immunometabolism in fibrosis. Other microRNAs, such as *miR-21*, *miR-199a-5p*, and *let-7d*, also play roles in fibrotic AM regulation. let-7c mitigates M1 phenotype and promotes the M2 phenotype [118]. *MiR-21* and *miR-155* are profibrotic, while *let-7i*, *miR-107*, *mir-126*, *miR-140*, and *miR-511* are antifibrotic [95]. *MiR-142-5p* increased in IPF and *MiR-155* exacerbates pathogenic PF, while *miR-140* protects against radiation-induced fibrosis. Non-coding RNAs modulate macrophage plasticity in fibrotic diseases, emphasizing the role of macrophage-derived microRNAs in PF [54,95].

Deciphering the complex epigenetic terrain of lung fibrosis is vital for pinpointing potential therapeutic targets. The reversibility of epigenetic modifications offers promise for drug development in fibrotic diseases. However, continued research is necessary to fully comprehend the intricate interplay of epigenetic mechanisms in AMs and their connection to lung fibrosis.

### 5.2. Chronic Obstructive Pulmonary Diseases

Progressive respiratory disease COPD is characterized by persistent airflow limitation and difficulties in breathing, incorporating chronic bronchitis and emphysema [119,120]. Among various etiological factors, cigarette smoking is a major cause for COPD occurring and induces a persistent epigenetic reprogramming of AMs [109]. The pathogenesis of COPD involves chronic airway inflammation, damage from oxidative stress, and an imbalance in protease/antiprotease mechanisms, with immunomodulatory disorders contributing to its development. Various immune cells, such as macrophages, neutrophils, and dendritic cells, play crucial roles in the immune functions associated with COPD [121]. Studies indicate a significant increase in macrophage numbers in COPD-affected areas, correlating with disease severity [17,19,121]. AMs in COPD are replenished through local proliferation and blood monocyte recruitment [122]. Recent research identified four macrophage phenotypes in COPD: non-polarized M0, iNOS-positive inflammatory M1, arginase-positive anti-inflammatory M2, and a dual positive-M1-M2 type, with a dominance of pro-inflammatory M1 macrophages in the small airways, while M2 macrophages, including subtypes M2a, M2b, M2c, and M2d, possess anti-inflammatory properties, promoting Th2-type immunity [121,123].

#### 5.2.1. DNA Methylation

Cigarette smoke causes genome-wide DNA methylation changes in AMs [100]. Notably, smokers’ AMs exhibit altered 5-methylcytosine and 5-hydroxymethylcytosine patterns at thousands of loci, leading to aberrant gene expression. Isolated DNA of AMs from heavy smokers and never smokers showed 25,000 loci of methylation status, indicating differential methylated genes ranges from immune system to inflammatory pathways, indicating a crucial role of methylation with CpG methylation associated with COPD disease occurrence and severity [27,124]. Changes in these DNA methylation patterns in COPD patients affect pro-inflammatory gene promoters in alveolar cells [124]. Among numerous genes, sphingosine-1-phosphate receptor 5 (*S1PR5*) encodes a G protein-coupled receptor and binds the lipid-signaling molecule sphingosine 1-phosphate in AMs and is known for regulating lung function and efferocytosis process [125,126]. Despite this, the increased expression of *S1PR5* in AMs of COPD patients showed reduced methylation associated with defective efferocytosis processes. Furthermore, several genes of lung macrophages, including *HSH2D*, *SNX10*, *CLIP4*, and *TYKZ*, are among 95 CpG loci with significant differences of methylation in COPD patients [127].

Furthermore, an epigenome-wide study of bronchoalveolar lavage cells found numerous differentially methylated CpG sites in smokers and COPD patients, including genes in the aryl hydrocarbon receptor pathway. These studies reveal differentially methylated genes, such as *SERPINA1* and *AHRR*, associated with COPD. Aberrant DNA methylation in genes like *GABRB1* and *TNFAIP2* is also observed in smokers and COPD [128,129]. Hyper- and hypomethylation in various genes are linked to COPD development, lung function decline, and metabolic differences in lung macrophages [38,130]. Overexpression of DNA methyltransferases correlate with abnormal DNA methylation, and exposure to cigarette smoke condensates affects their expression. Additionally, altered DNA methylation is observed in genes related to extracellular matrix proteins that support cells in tissue [131]. Hypomethylation of HDAC6 is reported, leading to HDAC activity and epithelial dysfunction. DNA methylation patterns in COPD patients in alveolar epithelial cells and AMs may affect the promoters of pro-inflammatory genes, potentially influencing macrophage function within the lungs [30,33].

#### 5.2.2. Histone Modification

Furthermore, COPD is strongly associated with an imbalance in histone acetylation homeostasis in AMs [132,133]. Oxidative stress from smoke inactivates histone deacetylases (especially HDAC2) in AMs, shifting the balance toward hyperacetylation of histones on promoters of inflammatory genes [133,134]. This acetylation-skewed state prolongs the transcription of cytokines, matrix metalloproteinases, and other damage mediators in COPD [135,136]. Indeed, HDAC2 is a key enzyme that normally deacetylates the chromatin at pro-inflammatory genes to shut down their expression; studies show that HDAC2 expression/activity is significantly reduced in COPD macrophages, which correlates with uncontrolled inflammation and steroid resistance [100,137]. In AMs from smokers, loss of HDAC2 activity leads to heightened production of IL-8, GM-CSF, and other cytokines despite corticosteroid treatment [138,139], fueling persistent airway inflammation. On the other hand, certain HDACs like HDAC3 can promote pro-inflammatory programs; interestingly, HDAC3 inhibition in COPD models has been suggested to reduce inflammation and favor an anti-inflammatory (M2) shift [100]. Thus, the pattern of histone modifications in COPD AMs is complex but generally favors prolonged inflammation through excess acetylation of inflammatory gene chromatin and selective methylation changes. Likewise, in DNA methylation, histone H3 phosphorylation is a key player in the transcription of *NF-κB* regulatory genes, crucial for the inflammatory response in COPD. Cigarette smoke induces histone H3 phospho-acetylation on pro-inflammatory gene promoters, emphasizing its importance in NF-κB transcription during exposure to cigarette smoke stimuli [140,141]. It has been known that HDAC is a key molecule in the repression of production of pro-inflammatory cytokines in AMs, and a decrease in HDAC is associated with increased inflammation in COPD and an increase in phosphorylated p38 MAPKs, playing a pivotal role in generating inflammatory cytokines in COPD smokers [142]. Reactive oxygen species, common in COPD, may also heighten inflammation by activating and phosphorylating MAPKs, suggesting smoke-induced COPD activate kinases, leading to histone phosphorylation and subsequent transcription of inflammatory genes, influencing the behavior of AMs. Targeting these kinases could be a potential therapeutic strategy for managing chronic inflammatory responses associated with COPD, specifically in AMs [17,120,121].

Additionally, though histone ubiquitination for H2A (K119) and H2B (K20) are known to play a role in genomic stability and transcriptional regulation, the specific impact on AMs in COPD remains an area requiring further investigation for a comprehensive understanding of the underlying mechanisms [38,48]. On another epigenetic front, histone methylation, involving modifications on arginine and lysine amino acids, has emerged as a crucial regulator of gene expression. Different methylation sites on histones, including H4K20, H3K79, H3K36, H3K27, H3K9, and H3K4, influence biological functions and cellular processes, and its implications in AMs warrant further exploration [143,144]. Furthermore, protein arginine methyltransferases (PRMTs), crucial for arginine methylation, contribute to various cellular processes in AMs, creating an epigenetic signature linked to gene expression. Hypoxic stimuli and oxidative stress, known factors in COPD development, impact PRMT levels in AMs [119,145].

#### 5.2.3. Non-Coding RNA

Next, epigenetic control in COPD AMs is also mediated by ncRNAs. Smoke and oxidant stress can alter miRNA expression profiles in AMs, which in turn modulate inflammatory signaling. In smoker and COPD samples, the proportion of M1 cells was lower compared with M0 and M2 cells [146]. Genes specifically upregulated in M1 cells were primarily associated with inflammation and immunity. Additionally, in M1 macrophages, the expression levels of *miR-30a-5p*, *miR-200c-3p*, *miR-20b-5p*, *miR-199b-5p*, and *miR-301b-3p* were lower than those in M0 [146,147]. In M2 macrophages, the expression levels varied, with higher expression in *miR-30a-5p* and *miR-20b-5p* and lower expression in *miR-200c-3p* and *miR-301b-3p* compared with M1 [148]. This suggests a potential role of microRNA in AMs in the context of COPD. For example, one study found that *miR-218* and *miR-124* were downregulated in COPD macrophages, leading to overexpression of MMP-2/-9 and enhanced elastin degradation [100]. More directly, *miR-146a*, an anti-inflammatory microRNA, is often upregulated in endotoxin-tolerant or smoke-exposed macrophages, serving as negative feedback on *NF-κB*, but its chronic upregulation may contribute to the impaired immune response in COPD [149]. Long non-coding RNAs are less studied in COPD AMs; however, lncRNA MEG3 has been implicated in regulating IL-1β and IL-6 via interaction with DNA methyltransferases in macrophages [150]. Overall, ncRNAs provide an additional layer of epigenetic-like control by targeting mRNAs or chromatin-modifying complexes, ultimately shaping the inflammatory phenotype of COPD AMs.

Recent technological advancements have greatly improved our understanding of AMs in various lung diseases. A key development has been the rise of single-cell omics technologies, such as single-cell RNA sequencing (scRNA-seq), which have revealed the heterogeneity within AM populations and identified disease-specific subtypes, particularly in conditions like COPD [151,152,153]. These studies have shown how distinct AM gene expression profiles are linked to functional dysregulation in COPD, including impaired lipid uptake and reduced angiogenesis [152]. This approach highlighted significant chromatin landscape differences, emphasizing the role of epigenetic regulation in driving macrophage plasticity and dysfunction in COPD. Similarly, a single-cell multi-omics approach was conducted to profile AMs subpopulation in COPD, integrating scRNA-seq and chromatin accessibility data to uncover disease-specific transcriptional and epigenetic signatures [154,155]. Their study demonstrated that COPD-associated AM subsets exhibit differential enhancer activity in genes involved in immune response regulation and tissue repair, linking epigenetic changes to altered AM function [155]. Collectively, this knowledge may open avenues for developing targeted therapies aimed at modulating histone modifications to alleviate inflammation and improve the overall condition of individuals affected by COPD.

### 5.3. Pulmonary Arterial Hypertension

Pulmonary arterial hypertension (PAH), identified by increased blood pressure in the pulmonary arteries, entails complex interactions with resident macrophages, particularly AMs and IMs of the lungs [156,157]. These macrophages play a crucial role in orchestrating vascular remodeling, particularly in response to injury, inflammation, or hypoxia [1,158]. One of the central mechanisms by which AMs exert this influence is through the secretion of cytokines that not only activate traditional signaling pathways but also induce epigenetic reprogramming in vascular endothelial and smooth muscle cells [159,160,161]. These macrophages play a substantial role in the pathogenesis of PAH by establishing an inflammatory environment vascular remodeling [156,162]. Various key cytokines released by macrophages include TNF-α, IL-6, IL-1β, TGF-β, and VEGF, as well as chemokines like CXCL12 [160,161]. Through the release of pro-inflammatory cytokines like IL-6 and TNF-α, they contribute to vascular dysfunction and remodeling. These molecules engage their respective receptors on vascular cells, activating intracellular cascades such as the *NF-κB*, *STAT3*, and *SMAD* pathways, which in turn modulate the activity of epigenetic enzymes [161]. Furthermore, macrophages influence the proliferation and migration of vascular smooth muscle cells, contributing to pulmonary artery narrowing and increased vascular resistance in PAH through vascular remodeling [161,162]. Additionally, they play a role in endothelial dysfunction, a hallmark of PAH, and their dysregulated immune response sustains inflammation and drives vascular changes [160,161].

#### 5.3.1. DNA Methylation

Although concrete data are currently insufficient to firmly establish an epigenetic role of AM in PAH, recent findings highlight that altered DNA methylation levels at specific gene loci such as *superoxide dismutase 2* and *granulysin*, variations in histone H1 levels, aberrant expression of HDACs and bromodomain-containing protein 4, and disruptions in microRNA networks contribute to the emerging role of epigenetics in PAH [58,163]. Notably, studies indicate that vascular cells, including pulmonary artery endothelial cells, smooth muscle cells, and adventitial fibroblasts isolated from hypertensive lung vessels maintain a hyper-proliferative, apoptosis-resistant phenotype ex vivo, suggesting heritability and contributing to PAH progression [38,163]. The intersection with AMs in this context adds a layer of complexity, as macrophages are known for their involvement in inflammation and the vascular remodeling of key aspects of PAH pathology [164]. Additionally, macrophage-derived cytokines like TGF-β modulate DNA methylation patterns [161,165]. TGF-β activates SMAD-dependent pathways in endothelial and smooth muscle cells, upregulating DNA methyltransferases such as DNMT1 and DNMT3a [166,167]. This results in the hypermethylation of promoters of genes that typically inhibit fibrosis and proliferation, including SMAD7 and RASSF1A, thereby repressing their expression and promoting a vascular remodeling phenotype in PAH [167,168]

#### 5.3.2. Histone Modification

Histone post-translational modifications serve as crucial regulators of chromatin modification processes. Among these modifications, core histones lysine acetylation tightly controlled the opposing actions of HATs and HDACs [41,169]. Among various cytokines released from AMs in PAH, TNF-α and IL-6 activate the NF-κB and STAT3 pathways, respectively [170,171]. NF-κB, once translocated to the nucleus, recruit HATs such as p300/CBP to promoters of pro-angiogenic and matrix-remodeling genes like MMP9 and VEGF [170]. This leads to increased H3K27 acetylation, an epigenetic mark associated with active transcription, thereby enhancing expression of these genes and promoting vascular permeability and extracellular matrix breakdown [172,173]. Concurrently, IL-6-driven *STAT3* activation has been shown to recruit *EZH2*, a histone methyltransferase, to the promoters of anti-angiogenic genes such as *THBS1*, leading to trimethylation of H3K27 and transcriptional silencing of these inhibitory signals [174,175,176]. This shift in gene expression promotes angiogenesis, endothelial proliferation smooth muscle cell proliferation, and vascular remodeling. Importantly, the dysregulation of HDAC activity has emerged as a significant player in the pathogenesis of PAH, and a study found promising therapeutic outcomes using small-molecule HDAC inhibitors in diverse animal models of PAH [167]. HDAC inhibitors, recognized for their ability to impede cell proliferation and induce apoptosis in transformed cells, have shown therapeutic promise in preclinical studies [177]. These inhibitors effectively dampen inflammation, mitigate fibrosis, and inhibit restenosis, showcasing their efficacy in animal models of left ventricular dysfunction and in the treatment of PAH [178].

#### 5.3.3. Non-Coding RNA

Next, numerous studies have identified distinct expression patterns of miRNA in PAH lung samples and associated cells. In lung PAH tissue, *miR-29*, *miR-17*, and *miR-223* showed high expression, while miR-1, miR-124, miR-130, miR-138, miR-140, and miR-210 were downregulated [147,179]. In PAH smooth muscles, *miR-204* and *miR-29* were downregulated, and *miR-424* was upregulated [147,180]. Likewise, PAH epithelial cells exhibited upregulation of *miR-140*, and *miR-21*, with *miR-301* showing lower expression compared with healthy controls [180]. This suggests a potential role of microRNA in PAH. Furthermore, induction of cytokine signaling in PAH can also influence non-coding RNAs. For example, IL-1β and TNF-α upregulate *miR-155*, which targets negative regulators of inflammation and angiogenesis, further reinforcing the remodeling process [181,182]. Collectively, these epigenetic changes induced by AM-derived cytokines establish a permissive transcriptional landscape in vascular cells, driving structural and functional alterations characteristic of pathological vascular remodeling [183,184,185].

Despite the acknowledged role of AMs and miRNA in PAH, none of the research, to the best of our knowledge, has explored the intricate role of miRNA within AM to PAH. Understanding the epigenetic modulation of AMs could provide crucial insights into the intricate mechanisms driving PAH.

### 5.4. Lung Cancer

Lung cancer stands as a global health threat, claiming the highest mortality rates among malignancies. The intricate interplay between inflammation and tumor progression is a pivotal factor in the development of lung tumors. In lung cancer, non-small-cell lung carcinoma AMs in particular differentiate into tumor-associated macrophages (TAMs) in the tumor microenvironment, exhibiting diverse effects on the growth, progression, and metastasis of lung tumors [17,91,186]. The dichotomy of TAMs manifests through two distinct phenotypes, the M1 and M2 macrophages, each exerting contrasting influences on neoplasm advancement. TAMs are often immunosuppressive (like M2-polarized macrophages) and facilitate tumor growth, angiogenesis, and metastasis. Their pro-tumoral versus anti-tumoral functions are heavily influenced by epigenetic modifications [104]. Unlike genetic mutations in cancer cells, TAMs undergo mainly epigenetic reprogramming in response to tumor-derived signals [104]. The dynamic functionality of TAMs, shaped by cytokines, chemokines, cell interactions, and signaling pathways, underscores their potential as therapeutic targets in non-small-cell lung cancer [187]. Within the intricate context of lung cancer, AMs, pivotal components of the lung’s immune milieu, contribute to lung cancer epigenetics through various mechanisms. A wealth of studies underscores the pivotal role of M2 in fostering an environment conducive to lung cancer progression. M2 macrophages accomplish this by releasing a spectrum of chemokines and growth factors, including TGF-β, IL-10, and MMP, fueling the growth, invasion, immunosuppression, and metastasis of lung cancer [17,91,187]. In addition, DNA methylation alterations in macrophages may influence the regulation of tumor suppressor genes, potentially affecting their anti-tumor functions and contributing to an immunosuppressive microenvironment that facilitates cancer progression [188,189].

#### 5.4.1. DNA Methylation

Lung cancer exhibits altered methylation in various oncogenes, silencing their expression primarily through hypermethylation of CpG islands in their promoter regions. DNA methylation likely silences certain genes in TAMs that would otherwise attack tumor cells. The hypoxic, cytokine-rich TME can induce DNMTs in TAMs, leading to hypermethylation of promoters for M1-associated genes including *IL12B* or *CXCL9* and other checkpoints that activate T cells. One study noted that tumor-derived lactic acid altered the DNA methylation pattern of oxidative metabolism genes in M1 macrophages, pushing them towards an M2 state [104]. Consistently, inhibiting DNMTs can skew macrophages toward a more inflammatory phenotype. For instance, blocking DNMT activity prevented tumor-induced metabolic reprogramming in macrophages in vitro [104,115,190]. However, direct analyses of TAM-specific DNA methylation remain sparse, and more profiling is needed. It is generally acknowledged that using DNMT inhibitors in cancer therapy can rejuvenate immune responses in the TME, partly by re-activating silenced immune genes in both tumor cells and infiltrating macrophages [104,191,192,193]. In summary, DNA methylation in TAMs contributes to immune evasion by repressing genes for antigen presentation and pro-inflammatory cytokines, but it may be reversible by epigenetic therapy.

*EZH2* is a key component of the polycomb repressive complex 2 (*PRC2*), which plays a crucial role in regulating gene expression by modifying chromatin structure. As a histone methyltransferase, *EZH2* plays a pivotal role in regulating cell cycle, autophagy, and apoptosis, particularly in cancer. Stimulation of TAMs showed *PRMT1*-mediated *EZH2* methylation, promoting lung cancer proliferation and metastasis by modulating oncogene methylation through *EZH2* [189,194]. Notably, hypermethylation of promoter regions in tumor suppressor genes is a recurring theme, resulting in the silencing of genes that normally act as safeguards against uncontrolled cell growth. Concurrently, hypomethylation in specific genomic regions, including oncogenes, fuels their overexpression, promoting cell proliferation and contributing to the oncogenic milieu [195]. Beyond diagnostics, the understanding of these methylation changes has opened avenues for epigenetic therapies [34,93,196]. Exploration of DNA methyltransferase inhibitors as potential interventions is underway to reverse aberrant DNA methylation and reactivate silenced tumor suppressor genes. The observed epigenetic heterogeneity in lung cancer, characterized by distinct DNA methylation profiles among subtypes, underscores the complexity of the disease and underscores the necessity for tailored therapeutic approaches [197,198].

#### 5.4.2. Histone Modification

Moreover, histone modifications within macrophages play a crucial role in shaping their immune response [43,45]. Changes in histone acetylation and methylation patterns can influence the pro-inflammatory or anti-inflammatory phenotype of macrophages, impacting their ability to recognize and eliminate cancer cells [189]. Unraveling the specific contributions of macrophage-associated epigenetic changes in lung cancer progression holds promise for developing targeted therapeutic strategies that leverage the interplay between the immune system and the epigenetic landscape of cancer cells [189,199]. Furthermore, TAMs exhibit characteristic histone modification patterns that promote an anti-inflammatory, pro-tumor phenotype. IL-4 and IL-10 in the TME upregulate JMJD3 in TAMs, as mentioned earlier, which removes the H3K27me3 repressive marks from M2 genes (e.g., *ARG1*, *CHI3L1*) and from genes that inhibit inflammation (like *IL1RA*) [104]. The same mechanism is operative in tumors: high JMJD3 levels in TAMs correlate with enhanced expression of M2 markers and immunosuppressive factors. On the other hand, certain histone methyltransferases can restrain macrophage activation. For example, SUV420H1-mediated H4K20 trimethylation and SMYD2-mediated H3K36 dimethylation set repressive checkpoints on TLR4-inducible genes in macrophages [104]. If tumor signals downregulate these enzymes, TAMs might overproduce inhibitory cytokines like IL-10. Additionally, histone acetylation is widely modulated in TAMs: tumor TAMs often have low expression of class II HDACs (e.g., *HDAC9*, *HDAC10*), leading to hyperacetylation and activation of genes that paradoxically encourage macrophages to be trophic rather than toxic to tumors. Notably, *HDAC11* stands out—it is a negative regulator of IL-10 in macrophages. Tumor exosomes containing *miR-145* have been shown to downregulate *HDAC11* in TAMs, unleashing IL-10 production and thereby promoting tumor immune evasion [104]. Neutralizing exosomal *miR-145* or inhibiting *IL-10* signaling can reduce tumor growth in such models [104]. Moreover, TAMs in many cancers exhibit increased histone lactylation due to abundant tumor cell-derived lactate. Lactylation of histone H3 induces transcription of *Arg1* and *VEGF* in TAMs, reinforcing their tissue repair, pro-angiogenic program. Overall, tumors create an epigenetic milieu (via cytokines, exo-metabolites, and exosomes) that re-wires histone marks in AMs/TAMs to favor tumor progression.

Recent clinical trials have explored the effects of HDAC and DNMT inhibitors on AM function, particularly in the context of lung cancer. A Phase II trial combined the DNMT inhibitor 5-azacitidine with the HDAC inhibitor entinostat in patients with advanced non-small-cell lung cancer. This combination aimed to reverse the epigenetic silencing of tumor suppressor genes [200,201]. Another Phase I clinical trial investigated the combination of panobinostat, a pan-HDAC inhibitor, with PDR001, an immune checkpoint inhibitor, in patients with advanced non-small-cell lung adenocarcinoma. This study explored the potential of combining epigenetic modulation with immune checkpoint blockade to enhance anti-tumor immunity [202,203,204]. Beyond clinical trials, preclinical research has demonstrated that combining DNMT and HDAC inhibitors can modulate macrophage responses. A study combining 5-azacitidine and trichostatin A in a mouse model of acute lung injury showed reduced inflammation and apoptosis, suggesting a potential therapeutic approach for conditions involving macrophage-mediated lung injury [205]. Furthermore, dual inhibitors targeting both DNMT and HDAC pathways have been developed for cancer therapy. Compound (R)-23a, a dual DNMT1/HDAC inhibitor, has shown potent inhibition of both targets, effectively reactivating tumor suppressor genes and suppressing tumor growth in xenograft models [206]. Another compound, C02S, demonstrated strong inhibitory activity against *DNMT1*, *DNMT3A*, *DNMT3B*, and *HDAC1*, leading to apoptosis, cell cycle arrest, reduced angiogenesis, and decreased tumor proliferation in vitro and in vivo [207]. These findings underscore the potential of dual epigenetic inhibitors in reshaping macrophage function and improving therapeutic outcomes in lung cancers and inflammatory lung conditions.

#### 5.4.3. Non-Coding RNA

Next, TAMs are heavily influenced by ncRNA-mediated regulation. Tumors secrete exosomes packed with microRNAs that enter macrophages and modulate their epigenetic and signaling pathways. We saw the example of miR-145 downregulating HDAC11 [104,208]. Another example is presented here: miR-138-5p delivered by tumor exosomes was found to target *KDM6B* (*JMJD3*) in macrophages, thereby upregulating H3K27me3 and blunting an M2-to-M1 reprogramming attempt [209]. Indeed, epigenetic therapies aimed at TAMs—such as combined DNA demethylating agents and HDAC inhibitors—have shown success in mouse models by disrupting immunosuppressive pre-metastatic niches and improving survival [210].

## 6. Chromatin Remodeler and Transcription Factor in the Epigenetic Regulation of AMs

To fully understand how epigenetic regulation shapes AM behavior, particularly in disease contexts, it is essential to consider the role of transcription factor (TF)-based epigenetic regulators, which modulate gene expression by altering chromatin structure without changing the underlying DNA sequence. These factors help control various processes such as development, differentiation, immune responses, and disease progression, including in lung diseases. Among various key epigenetic chromatin remodelers in regulating AMs function, particularly in the context of various lung diseases, bromodomain and extra-terminal (BET) proteins, including BRD2, BRD3, and BRD4, are critical for regulating the transcription of inflammatory genes [211,212]. These proteins recognize acetylated lysine residues on histones and non-histone proteins, facilitating the recruitment of transcriptional machinery to chromatin [213]. In diseases like COPD and lung disease conditions, BET proteins influence the expression of pro-inflammatory cytokines such as TNF-α, IL-6, and IL-1β [214,215]. Inhibition of BET proteins with small-molecule inhibitors, like JQ1, has shown promise in reducing inflammation and modulating macrophage polarization, suggesting potential therapeutic applications in pulmonary diseases [215]. Additionally, the BET inhibitor apabetalone was tested in the BETonMACE trial and showed cardiovascular and anti-inflammatory benefits [216,217]. Similarly, vorinostat, a histone deacetylase inhibitor (HDACi), has been investigated in early-phase trials for its capacity to suppress pro-inflammatory cytokine expression in pulmonary and fibrotic contexts [218]. While these agents show promising immune modulatory effects, several challenges limit their translational application to lung diseases. Among them is the issue of off-target effects, as most current epigenetic drugs act systemically and lack cell-type specificity, raising concerns about toxicity and global immune suppression. Additionally, effective delivery to AMs remains a significant barrier due to the anatomical complexity of the lung and the inaccessibility of these cells via traditional systemic routes [219]. Additionally, histone modifiers, such as HATs and HMTs, play pivotal roles in shaping macrophage gene expression. For instance, p300/CBP, major HATs, are involved in activating transcription by acetylating histones at the promoters of inflammatory genes [220]. G9a and SUV39H1, histone methyltransferases, modulate histone methylation, which in turn influences macrophage activation and polarization [221]. In diseases such as pulmonary fibrosis, aberrant histone modifications can drive chronic inflammation and fibrosis, underscoring the need for targeted therapies that modulate these chromatin modifiers [108]. In diseases like COPD, altered DNA methylation by DNMTs can affect immune gene expression, potentially impairing immune responses or promoting excessive inflammation [219].

Chromatin remodelers are protein complexes that regulate the accessibility of chromatin to transcriptional machinery, thereby influencing gene expression. In lung diseases, transcription factors like *NF-κB*, *AP-1*, and *IRF3* also play vital roles in regulating key genes in AMs. *NF-κB* is a central regulator of inflammation and is modulated by epigenetic changes such as histone acetylation [20,109]. *NF-κB* signaling contributes to the production of pro-inflammatory cytokines, while *AP-1* and *IRF3* regulate macrophage activation and immune responses to infection [77,170]. These TFs often interact with chromatin modifiers, affecting macrophage responses to environmental stimuli. The impact of these epigenetic factors varies across different conditions. In lung injury and chronic lung disease, the combined actions of transcription factors like *NF-κB* and *AP-1*, along with chromatin modifiers, regulate immune gene expression, leading to excessive inflammation [222]. Epigenetic therapies targeting these pathways could potentially reduce lung injury and promote tissue repair. In lung cancer, aberrant activation of BET proteins and altered DNA methylation can reshape the tumor microenvironment, with TAMs being reprogrammed to support tumor growth [212,214]. Targeting these pathways may reprogram TAMs from a pro-tumor to an anti-tumor phenotype, enhancing anti-cancer immunity. In pulmonary fibrosis, altered histone modification and DNA methylation contribute to fibroblast activation and excessive matrix deposition, and understanding how chromatin modifiers influence these processes in AMs may lead to strategies for reversing fibrosis. Furthermore, the therapeutic potential of epigenetic modifiers in treating chronic lung diseases such as COPD, pulmonary fibrosis, and PAH is increasingly recognized due to their capacity to reprogram macrophage phenotypes and attenuate pathogenic gene expression.

Technological advances, including CUT&RUN and single-cell RNA sequencing, have enabled precise profiling of transcription factors and histone modifications, providing new insights into how chromatin accessibility and metabolic states shape cellular identity and function within AMs [223]. Integration of multi-omics platforms offers a more comprehensive view of how environmental cues influence epigenetic regulation in lung health and disease [224]. Moreover, computational approaches—including machine learning—are increasingly being used to analyze these large datasets, identify novel AM subtypes, and predict disease-specific cellular behaviors. These technologies not only help uncover disease-associated macrophage subtypes but also facilitate the discovery of novel epigenetic biomarkers and therapeutic targets, offering a more detailed understanding of how the AM epigenome is reprogrammed during disease progression.

## 7. Microbial and Cellular Metabolites in the Epigenetic Regulation of AMs

Emerging evidence reveals that the gut microbiome profoundly influences the epigenetic landscape of AMs, offering new insights into the gut–lung axis [225,226]. Gut-derived short-chain fatty acids (SCFAs), such as butyrate, acetate, and propionate, act as signaling molecules that extend far beyond the intestinal environment [227]. Their capacity to inhibit HDACs is particularly noteworthy as HDACs are enzymes that remove acetyl groups from histone proteins, leading to chromatin condensation and gene silencing. By inhibiting HDACs, SCFAs promote histone acetylation, generally resulting in a more open chromatin structure and enhanced gene transcription [228]. In the context of AMs, the constant exposure to systemic SCFAs originating from the gut likely plays a role in shaping their baseline epigenetic profile during homeostasis [229]. This could influence the expression of genes critical for their sentinel functions, including efferocytosis, a process vital for maintaining alveolar integrity and preventing the release of pro-inflammatory contents. For instance, butyrate-mediated HDAC inhibition might upregulate genes involved in the recognition and engulfment of dead cells, contributing to tissue remodeling and resolution of minor injury in the lungs [230]. However, when the delicate balance of the gut microbiome is disrupted, the production of SCFAs can be altered, both in quantity and composition. This shift in the systemic availability of HDAC inhibitors can have profound consequences for the epigenetic programming of AMs. In diseases such as COPD [231], where altered gut microbiota profiles are commonly observed, reduced levels of SCFAs may lead to decreased histone acetylation in AMs. This epigenetic shift could promote a pro-inflammatory phenotype, increasing their responsiveness to allergens and contributing to airway hyperresponsiveness and inflammation. In COPD specifically, where impaired clearance of cellular debris and chronic inflammation are key features, dysbiosis-related changes in SCFA levels may further impair the efferocytosis capacity of AMs, exacerbating tissue damage and sustaining inflammation [227,228,232]. Understanding these intricate epigenetic mechanisms driven by gut microbial metabolites offers promising avenues for therapeutic interventions aimed at modulating the gut microbiome or directly targeting epigenetic pathways in AMs to restore lung health.

The epigenetic regulation of AMs is inherently dynamic and closely intertwined with cellular metabolism [67]. Beyond the influence of microbial-derived SCFAs, a wide range of endogenous metabolites act as direct regulators of the enzymes responsible for establishing and maintaining epigenetic marks [70,227]. These metabolites effectively connect the metabolic state of AMs to their gene expression patterns and functional outcomes. A central player in this metabolic–epigenetic interface is the one-carbon metabolism pathway, which includes folate and methionine cycles [233]. Methionine is converted into S-adenosylmethionine (SAM), the primary methyl donor used by DNMTs to add methyl groups to cytosine residues in DNA—a modification typically associated with gene repression. The availability of methionine, which is influenced by both diet and cellular metabolic activity, directly impacts SAM levels and thereby modulates DNA methylation patterns across the AM genome [234,235]. These changes can significantly affect the expression of genes involved in inflammation, phagocytosis, and tissue repair. Similarly, the tricarboxylic acid (TCA) cycle, a key source of cellular energy metabolism, produces various intermediates that serve as cofactors or inhibitors for histone-modifying enzymes [236]. For example, α-ketoglutarate is an essential co-substrate for Jumonji C domain-containing histone demethylases (KDMs), which remove methyl groups from lysine residues on histones [237]. This activity typically leads to a more relaxed chromatin structure and promotes gene transcription. Alterations in glucose and glutamine metabolism can influence intracellular α-KG levels, thereby modulating KDM activity and shaping chromatin accessibility in AMs in response to various stimuli [65]. Conversely, TCA cycle intermediates like succinate and fumarate can act as inhibitors of specific histone demethylases and methyltransferases [238]. Under inflammatory conditions, succinate can accumulate and inhibit KDMs, resulting in increased histone methylation and a transcriptional profile skewed toward pro-inflammatory responses [239]. Meanwhile, fumarates modify cysteine residues on proteins including epigenetic enzymes to alter their function [238]. Another key metabolite, acetyl-CoA, produced from glucose and fatty acid metabolism, serves as the primary acetyl donor for HATs [72]. The availability of acetyl-CoA directly influences global levels of histone acetylation, a modification generally linked to transcriptional activation [65,240]. Shifts in fuel utilization by the cell can rapidly affect histone acetylation patterns, enabling AMs to swiftly adjust gene expression in response to environmental cues.

In essence, cellular metabolism acts as a finely tuned regulatory layer over the alveolar macrophage epigenome. By sensing and integrating metabolic signals, AMs can adapt their transcriptional programs to meet the diverse demands of the lung microenvironment, whether it is clearing apoptotic cells during homeostasis or mounting robust immune responses during infection or inflammation [240,241]. Unraveling these metabolic–epigenetic interactions hold great promise for developing therapies that target macrophage function by modulating metabolic pathways or epigenetic enzymes.

## 8. Targeting Macrophages as a Therapeutic Approach

The therapeutic targeting of macrophages in lung diseases has gained substantial attention, given their crucial role in both lung health and disease. Whether they are addressing infectious lung diseases like COPD [120], pulmonary fibrosis [86], PAH [167], or lung cancer [189], macrophages intricately contribute to lung immunity, inflammation, and tissue repair. Strategies for infectious diseases may involve enhancing macrophage function for effective pathogen combat or modulating responses to mitigate excessive inflammation. In pulmonary fibrosis, directly targeting pro-inflammatory monocyte-derived AMs show promise in preventing fibrosis progression [91]. Balancing pro-inflammatory and anti-inflammatory macrophages is explored in COPD and PAH. Targeting macrophages in lung cancer involves reprogramming tumor-associated macrophages for anti-tumor effects and enhancing treatment efficacy [1,86,91,242]. Immunomodulation to transition macrophages to anti-inflammatory or tissue-repairing states is also considered.

Although HDAC and DNMT inhibitors are primarily studied for their roles in cancer and other conditions [243,244,245], but their potential impact on AM function presents a compelling area for further exploration. HDAC inhibitors work by preventing the deacetylation of histones and increased gene expression. In AMs, this may enhance inflammatory responses by promoting the expression of pro-inflammatory cytokines and chemokines, thus increasing macrophage activation [43,246]. Additionally, HDAC inhibitors may influence macrophage polarization, potentially skewing them towards a more pro-inflammatory or anti-inflammatory state depending on the specific context [246,247]. In lung diseases like COPD, these inhibitors might help resolve inflammation by promoting the production of anti-inflammatory mediators. In the case of lung fibrosis, HDAC inhibitors could reduce excessive collagen deposition by modulating the interactions between macrophages and fibroblasts [108,248]. DNMT inhibitors, on the other hand, prevent DNA methylation, which can reactivate previously silenced genes [249]. In AMs, this could lead to the reactivation of tumor suppressor genes or genes that regulate immune responses, potentially altering macrophage-mediated phagocytosis, cytokine production, or antigen presentation. In chronic inflammatory diseases like COPD or interstitial lung disease, DNMT inhibitors might modulate the expression of genes involved in inflammation [250,251].

Above all, macrophage-directed therapy, utilizing diverse approaches such as targeted drug delivery, immunotherapies, gene therapies, and combination strategies, presents a promising avenue for revolutionizing the management of complex lung diseases. However, a comprehensive understanding of their mechanisms and clinical efficacy requires further investigation. In the broader context, while the therapeutic potential of specific inhibitors within AMs is still under scrutiny, their combination with other immunotherapies holds the potential to unlock novel treatment strategies for conditions beyond lung disease, including lung cancer, infections, and autoimmune disorders.

## 9. Conclusions

In conclusion, AMs emerge as pivotal contributors in lung diseases, thus positioning them as promising targets for therapeutic interventions. Strategies range from enhancing their functional capabilities to modulating their responses, with tailored approaches being designed for specific lung conditions. These strategies harness the potential of AMs for the effective management of respiratory disorders. Importantly, epigenetics plays a central role as a key determinant in shaping these therapeutic avenues, further highlighting the intricate molecular mechanisms governing AM functions in the context of lung health and disease. The intricate interplay among methylation, histone modifications, microRNA, and histone variants, individually or through crosstalk, establishes a critical epigenetic relationship.

## Data Availability

No new data were created or analyzed in this study.
